# Wrist–ankle acupuncture attenuates cancer-induced bone pain by regulating descending pain-modulating system in a rat model

**DOI:** 10.1186/s13020-020-0289-y

**Published:** 2020-02-04

**Authors:** Chunpeng Zhang, Chen Xia, Xiaowen Zhang, Weimin Li, Xuerong Miao, Qinghui Zhou

**Affiliations:** 1 School of Traditional Chinese Medicine, Changhai Hospital, Naval Medical University, 168 Changhai Road, Shanghai, 200433 People’s Republic of China; 2 Laboratory of Neuronal Network and Systems Biology, Shanghai Medical College, Fudan University, Shanghai, 200032 China; 3 Department of Anesthesiology, Eastern Hepatobiliary Surgery Hospital, Naval Medical University, 225 Changhai Road, Shanghai, 200433 People’s Republic of China

**Keywords:** Wrist–ankle acupuncture, Electroacupuncture, Cancer pain, Descending modulation

## Abstract

**Background:**

Cancer-induced bone pain (CIBP) presents a multiple-mechanism of chronic pain involving both inflammatory and neuropathic pain, and its pathogenesis is closely related to endogenous descending system of pain control. However, the action mechanism underlying the effects of wrist–ankle acupuncture (WAA) versus electroacupuncture (EA) on CIBP remains unknown.

**Methods:**

Thirty-two Wistar rats were divided into sham, CIBP, EA-treated and WAA-treated groups. CIBP was induced in rats of the latter three groups. Time courses of weight and mechanical hyperalgesia threshold (MHT) were evaluated. After 6 days of EA or WAA treatment, the expressions of 5-hydroxytryotamine type 3A receptor (5-HT_3A_R) and *μ*-opioid receptor (MOR) in rostral ventromedial medulla (RVM) and/or spinal cord, as well as the levels of 5-HT, β-endorphin, endomorphin-1 and endomorphin-2 in RVM and spinal cord, were detected.

**Results:**

Injection of cancer cells caused decreased MHT, which was attenuated by EA or WAA (*P* < 0.05). WAA had a quicker analgesic effect than EA (*P* < 0.05). No significant difference of MOR in RVM was found among the four groups. EA or WAA counteracted the cancer-driven upregulation of 5-HT_3A_R and downregulation of MOR in spinal cord (*P* < 0.05), and upregulation of 5-HT and downregulation of endomorphin-1 in both RVM and spinal cord (*P* < 0.05). β-endorphin and endomorphin-2 in RVM and spinal cord decreased in CIBP group compared with sham group (*P* < 0.05), but EA or WAA showed no significant effect on them, although a tendency of increasing effect was observed.

**Conclusion:**

WAA, similar to EA, alleviated mechanical hyperalgesia in CIBP rats by suppressing the expressions of 5-HT and 5-HT_3A_R, and increasing the expressions of MOR and endomorphin-1 in RVM-spinal cord pathway of the descending pain-modulating system. However, WAA produced a quicker analgesic effect than EA, the mechanisms of which need further investigation.

## Background

Cancer-related pain, caused by bone metastasis from malignant tumors, is the most common and extremely devastating symptom that decreases quality of patients’ life; more than 70% of patients with advanced cancer have suffered from cancer-induced bone pain (CIBP) [1, 2]. Opioids remain the mainstay of clinical drug treatment for CIBP, often in combination with initial radiotherapy and adjuvant agents inhibiting osteoclast activity [3–6]. However, only half patients with CIBP gain temporary pain relief from conventional analgesics, and new and better therapies for CIBP alleviation with few adverse effects are urgently required [7, 8].

Wrist–ankle acupuncture (WAA) is a modern acupuncture therapy performed through the subcutaneous insertion of needles at points on the wrist and ankle regions. WAA theory is quite different from that of traditional acupuncture, and this treatment does not induce any pain or “needling sensation”, an unpleasant feeling such as local fullness, numbness, and soreness [9–11]. Due to its wide range of analgesic effects for various pain states, WAA is easily accepted by patients with cancer-related pain. Previously, our team examined the analgesic efficacy of WAA for patients with hepatocarcinoma-induced pain, and its analgesic mechanism has also been discussed in our series of studies [12–15].

CIBP presents a multiple-mechanism of chronic pain involving both inflammatory and neuropathic pain, and often occurs at more than one site due to tumor expansion, causing damage to the primary afferent nerve fibers of bone and releasing various inflammatory mediators [16, 17]. The rostral ventromedial medullar (RVM) in brain plays a crucial role in the endogenous descending system of pain control, exerting pain inhibitory/facilitatory actions [18–20]. Previous evidence has suggested that the predominant RVM descending modulating system impacts central sensitization and magnification of the pain response in the spinal cord when chronic pain persists [21–23].

Electroacupuncture (EA) has been found to be effective for relieving various pain states including cancer pain, and its analgesic effect on animal with CIBP has been examined in recent studies [24, 25]. EA with lower-frequency (2 Hz) stimulates β-endorphin (β-EP) and endomorphin (EM) release, which activates *μ*-opioid receptor (MOR) in brain, and EA with higher-frequency (100 Hz) stimulates dynorphin, which activates MOR in the spinal cord [26–28]. Combination of these two frequencies results in the simultaneous release of all these substances, leading to a maximal analgesic efficacy. Several studies have suggested that the analgesic mechanism of EA might be connected with the inhibitory/facilitatory descending system of pain, especially with MOR and 5-hydroxytryotamine (5-HT) in the RVM-spinal cord pathway [29–32]. However, no animal experiments studying the mechanism of WAA for regulating CIBP have been performed to date.

Stimulation with WAA is quite different to EA stimulation, and it is unknown whether the mechanism of WAA is similar or different to that of EA. We hypothesized that WAA, as EA, would inhibit CIBP by mediating the descending pain inhibitory/facilitatory system. Therefore, in this study, by establishing a CIBP rat model, we investigated the effect of WAA on CIBP by experimental methods such as praxeology and molecular biology and compared the analgesic mechanisms of WAA and EA on CIBP.

## Materials and methods

### Animals and grouping

Thirty-two specific pathogen-free grade female Wistar rats weighing 140–160 g for establishment of the animal model, and three weighing 70 g for ascites passage of tumor cells, were purchased from Shanghai Slac Laboratory Animal Co., Ltd. (Shanghai, China; No. SCXK[Hu]2007-0001). The animals were maintained at the Animal Experimentation Center of Changhai Hospital in a controlled environment of 20–24 °C, with a 50%–70% humidity range, a 12 h light/dark cycle, with food and water ad libitum. The study protocol and experiments were approved by the Animal Care and Use Committee of Naval Medical University. Walker 256 mammary cancer cells were obtained from Shanghai Biomedical Engineering Research Center.

After 3 days of adaptive feeding, the 32 rats for establishment of the animal model were randomly divided into four groups with eight rats in each group: sham, CIBP, CIBP plus EA, and CIBP plus WAA groups. Body weights were measured every day. All rats were euthanized on day 16 (D 16), and the sample of ipsilateral spinal cord and RVM were excised according to the rat brain atlas for further study [33]. Four rats in each group were randomly selected for molecular experiment, and the other 4 rats in each group were transcardially perfused with 4% paraformaldehyde for immunohistochemical staining. The sample of relevant tissues was diluted 10 times with PBS after homogenation for different experiments.

### Induction of bone cancer pain

Bone cancer pain was induced in rats according to the methods described in previous studies [34, 35]. Briefly, intraperitoneal injection of Walker 256 mammary cancer cells was performed on the rats, and then the ascites was collected on day 7 after injection. The cell density was adjusted to 1 × 10^5^/μL. Thermal-inactivated cancer cells were also prepared. After sterilizing the shaved left hind limb, the cancer cells (4 × 10^5^ cells in 10 μL) were injected into the medullary cavity of the left tibia through the tibial plateau using a microsyringe (10 μL; Hamilton Co, Bonaduz, Switzerland) to induce bone cancer in the rats, and the injection hole was quickly sealed with bone wax. Equivalent thermal-inactivated cancer cells were performed through a similar process in the sham group.

### Acupuncture treatments

For acupuncture treatments, Hwato brand disposable acupuncture needles (0.16 mm × 7 mm; Suzhou Medical Appliance Factory, Suzhou, China) and SDZ-V EA apparatuses (Nerve and Muscle Stimulator SDZ-V; Suzhou Medical Appliance Factory, Suzhou, China) were used.

**Fig. 1:**
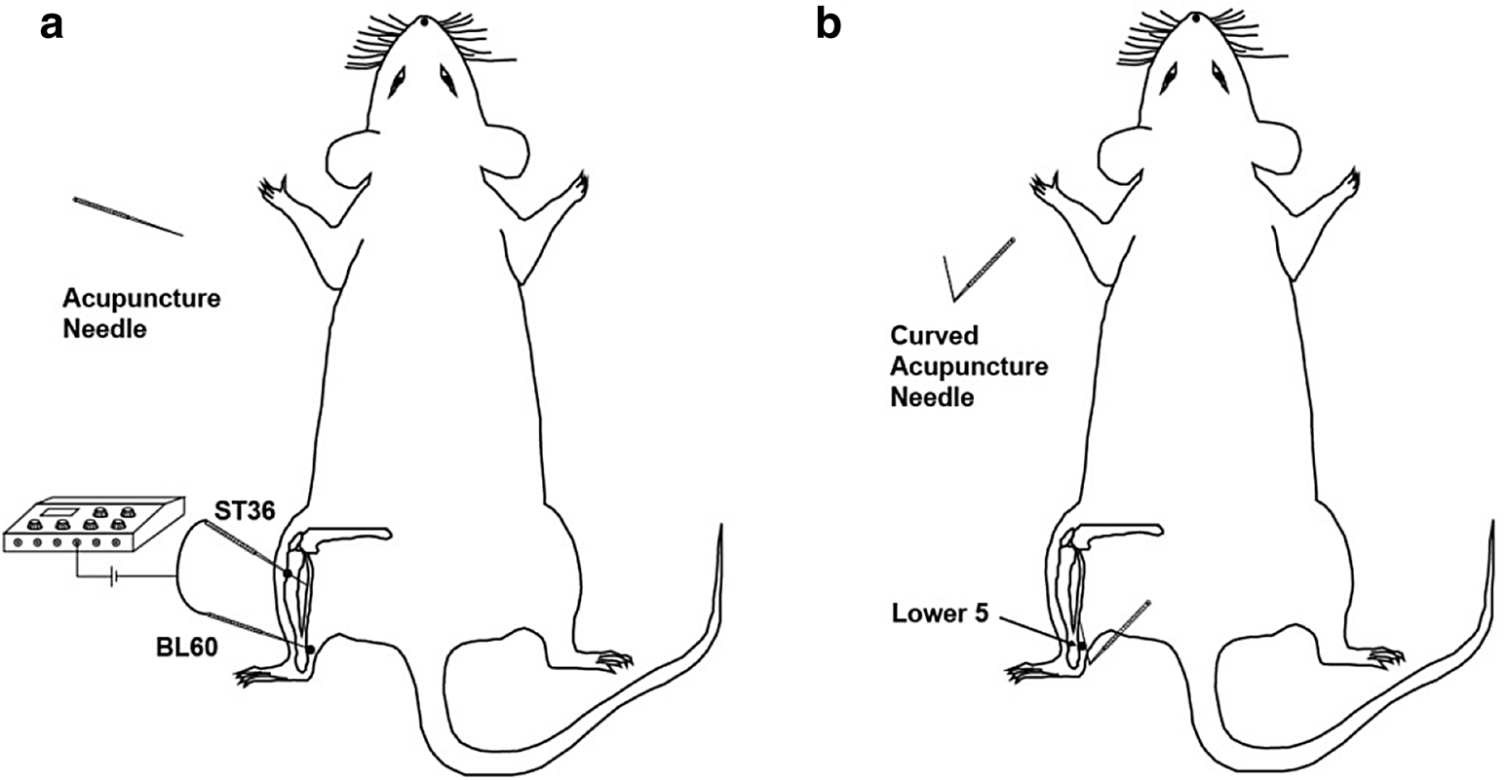
Needling of electroacupuncture (EA) and wrist–ankle acupuncture (WAA) at the hind limb. **a** Electroacupuncture of ST36 and BL60; **b** wrist–ankle acupuncture of Lower Point 5

Two acupoints, Zusanli (ST36, located at one-fifth point below and lateral to the anterior tubercle of the tibia) and Kunlun (BL60, located at the depression posterior to the lateral malleolus of tibiofibula), were commonly used for EA treatment of cancer pain in animal studies [32, 36] (Fig. 1a). In this study, rats in the EA group received acupuncture at left ST36 and BL60 with needle insertion approximately 4 mm into the skin (Fig. 1a). Paired electrodes from the EA apparatus were attached to the needle handles. The EA stimulation was applied for 30 min with a dense-disperse wave of 2/100 Hz and a current intensity of 2 mA.

In the WAA group, rats were needled at Lower Point 5 (the same location of BL60) at the left hind limb, subcutaneously towards the surgical site [10] (Fig. 1b). The needle remained with a tape in the superficial layer of the subcutaneous tissue until 8:00 the next day.

All treatments were started at 16:00–17:00 of day 10 after the induction of bone cancer pain, once a day for 6 continuous days.

Rats in the sham and CIBP groups did not receive acupuncture intervention. Rats in all four groups wore a self-made headgear from 16:00 to 8:00 the next morning to prevent biting [37]. In order to limit the rat activity during treatment proceeding, rats in all groups were fixed properly at the time of EA treatment.

### Measurement of pain behavior

**Fig. 2:**
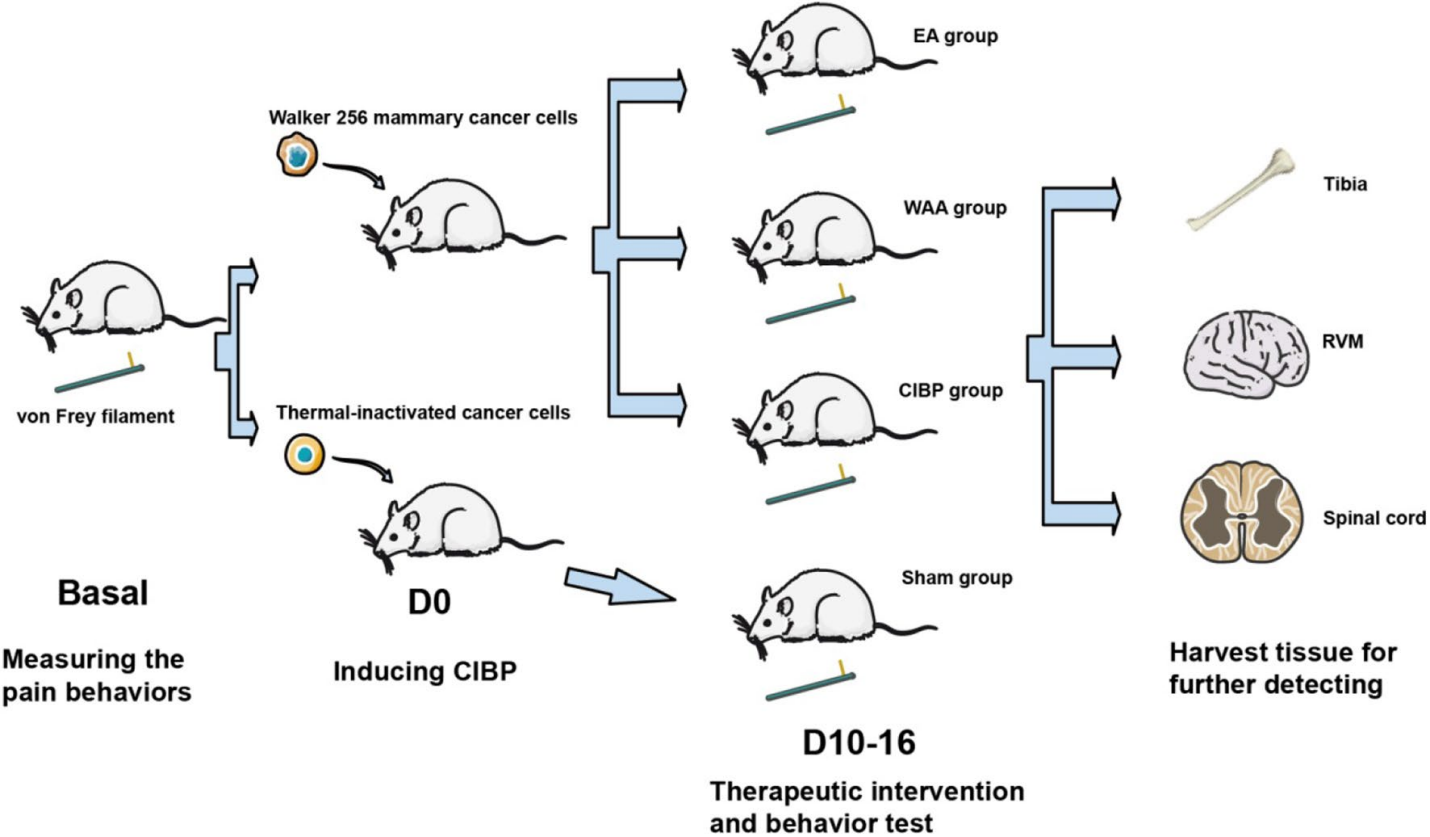
Experimental protocol for the assessment of pain. *CIBP* cancer-induced bone pain

The 50% paw withdrawal threshold (PWT) of the rats was measured using the up-down method of Dixon [38], and was performed during 10:00–14:00 before the injection of the cancer cells (baseline), at every other day after the injection (D1, D3, D5, D7, and D9), and during 17:00–18:00 after the treatments of each group, at every other day when intervention involved (D11, D13, and D15). The experimental process is shown in Fig. 2. For von Frey testing, a cellosilk probe called von Frey filament (Stoelting, IL, USA) was used to poke the hind paw pad with a sufficient upward force. A positive response was noted if the hind paw was sharply withdrawn, and the value of the final von Frey filament was recorded for calculation. The testing was repeated 8 times at 3-min intervals, and the target force values ranged 0.4–15 were used to prevent damage. 50% PWT value was conversed by the following formula [39]:

$$ 50{\%}\,  {\text {PWT}} = {{\left( {10^{{\left[ {X_{f} + k{{\delta }}} \right]}} } \right)} \mathord{\left/ {\vphantom {{\left( {10^{{\left[ {X_{f} + k{{\delta }}} \right]}} } \right)} {10,000}}} \right. \kern-0pt} {10,000}} $$

where *X*_*f*_  = value (in log units) of the final von Frey filament used; *k* = tabular value for the pattern of positive/negative responses; and δ = mean difference (in log units) between stimuli.

### Hematoxylin and eosin staining

For histopathological examination, left tibias were extracted at day 16 (D16), fixed in 10% neutral buffered formalin for 48 h, and then decalcified in ethylenediaminetetraacetic acid. Then the adequately decalcified tibias followed a regular hematoxylin and eosin staining process [40]: dehydration, transparency, embedding, slicing, incubating, dewaxing, and washing. These sections were stained with hematoxylin and eosin, and the slices were examined using light microscopy.

### Real-time polymerase chain reaction

The mRNA expression levels of 5-HT_3A_R and MOR within the spinal cord and RVM, were measured by real-time polymerase chain reaction (PCR) as described [41]. More specifically, samples were prepared by homogenization with TRIzol reagent (Invitrogen, Carlsbad, CA) and RNA was extracted according to the instructions. Next, reverse transcription (RT) was performed to convert RNA into cDNA using the RT premix kit (Thermo, Waltham, MA, US) with oligo DT primers. Real-time PCR was performed using 2 μL of cDNA with SYBR-Green PCR Mix plus (Thermo, Waltham, MA, USA). The primers for real-time PCR were as follows: MOR primer, 5′-ACCGTTTCCTGGCACTTC-3′ and antisense primer, 5′-GTATTAGCCGTGGAGGGATG-3′; 5-HT_3A_R primer, 5′-AAGAAGTGAGGT CGGACAAGAG-3 and antisense primer, 5′-GGCTGACTGCGTAGAATAAAGG-3′; Glyceraldehyde-3-phosphate dehydrogenase (GAPDH) primer, 5′-TGGGCAAGGTCATCCCA GAG-3′, and antisense primer 5′-GAGGCCATGTAGGCCATGAG-3′.

The standard cycling conditions were as follows: initial denaturation (95 °C for 3 min), followed with 40 cycles involving denaturation (95 °C for 15 s), annealing (60 °C for 60 s), and extension (60 °C for 15 s). Relative fold changes of gene expression were analyzed using the ΔΔCT method by ABI Prism 7300 SDS software, and the values are expressed as 2^−ΔΔCt^.

### Western blotting

Western blotting followed a previously described procedure [42]. In brief, spinal cord and RVM samples were lysed in lysis buffer containing a protease inhibitor cocktail (Jrdun Biotech, Shanghai, China). The proteins were separated on 10% SDS-polyacrylamide gels (Jrdun Biotech, Shanghai, China) and transferred to polyvinylidene difluoride (PVDF) membranes (Jrdun Biotech, Shanghai, China). The blots were probed with primary antibodies against MOR (rabbit anti-MOR; 1:1000; Abcam, Cambridge, Shanghai, China) or 5-HT_3A_R (rabbit anti-5-HT_3A_R; 1:200; Abcam, Cambridge, Shanghai, China), and horseradish peroxidase (HRP)-conjugated secondary antibody (goat anti-rabbit; 1:1000; Beyotime Biotech, Shanghai, China). According to the enhanced chemiluminescence (ECL) method, gray value was measured by Image J software with GAPDH as control.

### Immunohistochemical staining

Rats were euthanized with 4% paraformaldehyde perfused transcardially in PBS (Jrdun Biotech, Shanghai, China) at pH 7.4. The brain and lumbar spinal cord (L4-L6) were cut into 7 μm slices, which were immunohistochemically stained as previously described [20]. Images of the stained slices were captured under a phase-contrast microscope (200× magnification) and the images were saved in ProgRes Capture Pro 2.7 Image analysis software (Jenoptik, Germany). The areas of 5-HT_3A_R and MOR expression in these sections, which corresponded to the superficial part of the spinal cord and RVM, were selected by Image Pro Plus software to calculate the positive ratio (positive ratio = positive area/observed area).

### Enzyme-linked immunosorbent assay

Levels of 5-HT, β-EP, endomorphin-1 (EM-1), and endomorphin-2 (EM-2) were determined using a sandwich enzyme-linked immunosorbent assay (ELISA) system. Samples were prepared by homogenization of the spinal cord or RVM in PBS (pH 7.4, 6 °C), and then collected through centrifugation (2500×*g* for 20 min). The supernatants were collected, and antigen levels were measured using corresponding ELISA kits (R&D Systems, Minneapolis, MN, USA) following the instructions.

### Statistical analysis

All statistical analyses were performed using SPSS 21.0, and Graphpad Prism 6 was used to create graphs. All data were described as mean ± standard deviations. For 50% PWTs, the differences were examined by a two-way analysis of covariance with repeated measurements. For other data, one-way analysis of variance (ANOVA) followed by a Dunnett’s test was used for the comparison of groups. *P* value less than 0.05 was regarded as statistically significant.

## Results

### Weight of the rats

**Fig. 3:**
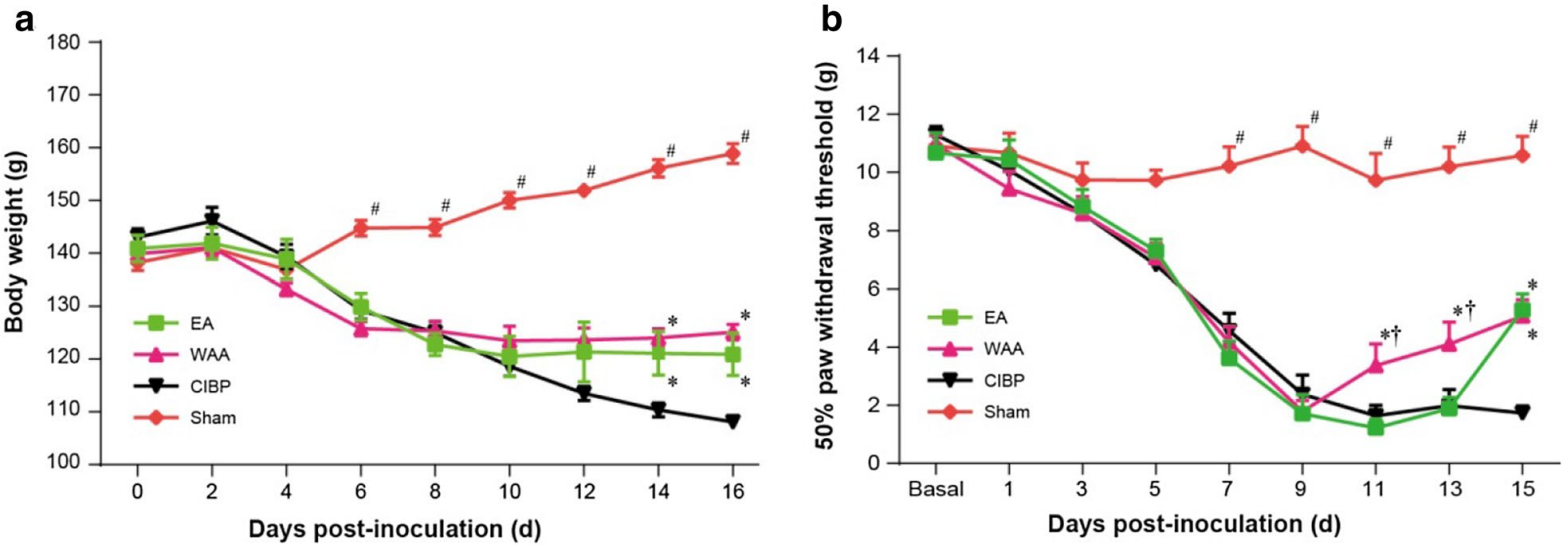
Effects of WAA on rat weight and ipsilateral 50% paw withdrawal threshold. **a** Effects of WAA on rat weight; **b** Effects of WAA on ipsilateral 50% paw withdrawal threshold. Values are mean ± standard deviations (*n* = 8). *P < 0.05 vs. CIBP group; ^#^*P *< 0.05 vs. model groups (CIBP, EA, and WAA groups). *CIBP* cancer-induced bone pain, *EA* electroacupuncture, *WAA* wrist–ankle acupuncture

Before EA and WAA intervention and on D6 after cancer cell injection, rats in the three model groups (CIBP, EA, and WAA groups) lost weight, and there were no significant differences in weight gain among the three groups. From D14 to D16, the rat weights of the EA and WAA groups were higher than that of the CIBP group, which showed no tendency to increase (*P *< 0.05, Fig. 3a). No significant differences in rat weights were observed between the EA and WAA groups throughout the experiment (*P *> 0.05, Fig. 3a).

### Mechanical hyperalgesia threshold

Figure 3b shows the effects of EA and WAA on 50% PWT of the ipsilateral hind paw of CIBP rats. Before inoculation of cancer cells (Basal), no significant differences were found in PWT among four groups (*P *> 0.05). The PWT in the three model groups (CIBP, EA, and WAA groups) decreased significantly from D7, with significant differences compared with the sham group (*P *< 0.05), but no significant differences were found among the CIBP, EA, and WAA groups on D7 and D9 (*P *> 0.05). On D11 and D13, WAA treatment significantly increased the PWT compared with the CIBP and EA groups (*P *< 0.05), indicating that WAA treatment could increase the mechanical hyperalgesia threshold (MHT). On D15, the PWT showed a significant increase in EA, but no difference found between the EA and WAA groups, indicating that WAA treatment produced a quick effect in increasing MHT and had a long-term effect equal to EA treatment.

### Observation of tibial tissue

**Fig. 4:**
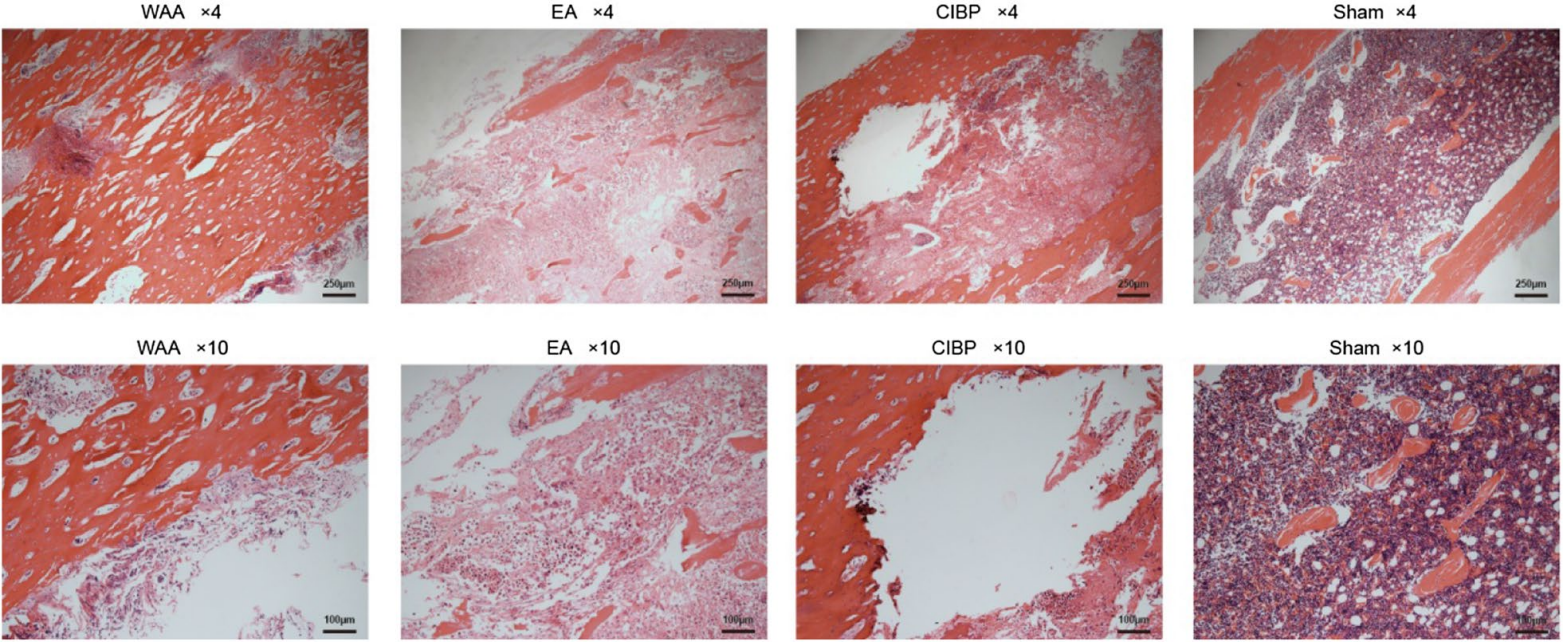
Tissue destruction of tibia in rats of the four groups stained with hematoxylin and eosin. *CIBP* cancer-induced bone pain, *EA* electroacupuncture, *WAA* wrist–ankle acupuncture

The hematoxylin and eosin-stained sections of the ipsilateral tibia from the sham group indicated healthy marrow without tumor growth and bone destruction. Sections of the ipsilateral tibias from all tumor cell-injected groups indicated abnormal polynuclear tumor cell replacement in the bone marrow cavity. The trabecular bones were widely eroded, and many braided bone formations were observed in the model group (Fig. 4).

### Analgesic mechanism of WAA

#### Effects of WAA and EA on 5-HT_3A_R mRNA and protein expression in the spinal cord

**Fig. 5:**
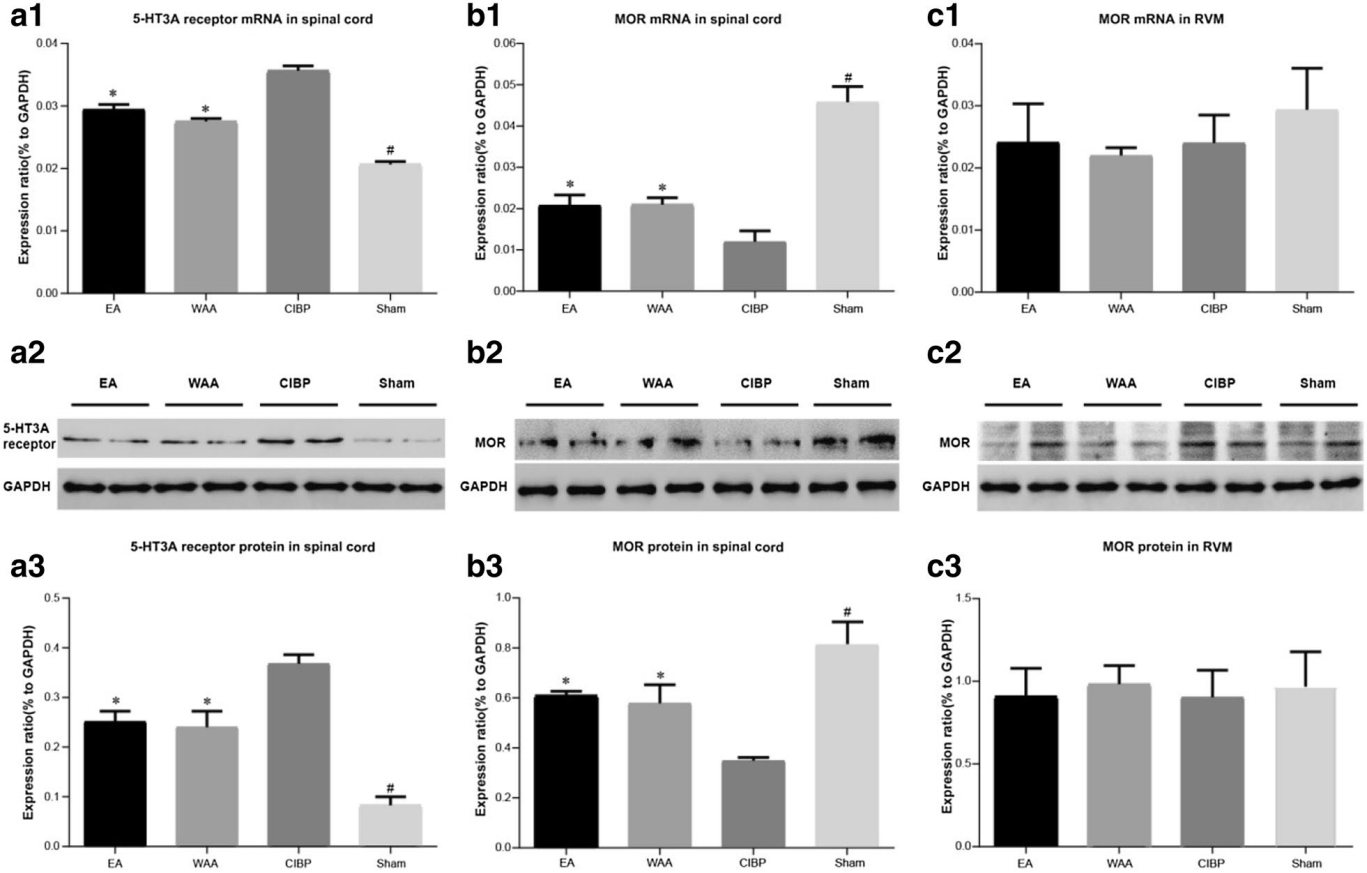
Effects of WAA on 5-HT_3A_R and MOR. Values are mean ± standard deviations (*n* = 4). *P < 0.05 vs. CIBP group; ^#^*P *< 0.05 vs. model groups (CIBP, EA, and WAA groups). *CIBP* cancer-induced bone pain, *EA* electroacupuncture, *WAA* wrist–ankle acupuncture, *5-HT*_*3A*_*R* 5-hydroxytryotamine type 3A receptor, *MOR* μ-opioid receptor, *RVM* rostral ventromedial medulla, *GAPDH* glyceraldehyde-3-phosphate dehydrogenase

In the CIBP, EA, and WAA groups, the levels of 5-HT_3A_R mRNA and protein in the corresponding segment of spinal cord were significantly higher than that in the sham group (*P *< 0.05). 5-HT_3A_R mRNA and protein levels were significantly lower in the EA and WAA groups than that in the CIBP group (*P *< 0.05), but no difference was found between these treatment groups (*P *> 0.05), as shown in Fig. 5a1–3. The results indicated that EA and WAA treatment could decrease the expression levels of 5-HT_3A_R in the spinal cord.

#### Effects of WAA and EA on MOR mRNA and protein expressions within the spinal cord and RVM

In the corresponding segment of spinal cord, there were significantly lower MOR mRNA and protein expressions (*P *< 0.05) in the three tumor-injected groups than in the sham group (*P *< 0.05), and more MOR mRNA and protein was found in the EA and WAA groups than in the CIBP group, but no significant difference was found between these two groups (*P *> 0.05), as shown in Fig. 5b1–3. But in the RVM (Fig. 5c1–3), the expression levels of MOR mRNA and protein showed no significant difference among four groups (*P *> 0.05).

#### Immunohistochemical results of 5-HT_3A_R and MOR

**Fig. 6:**
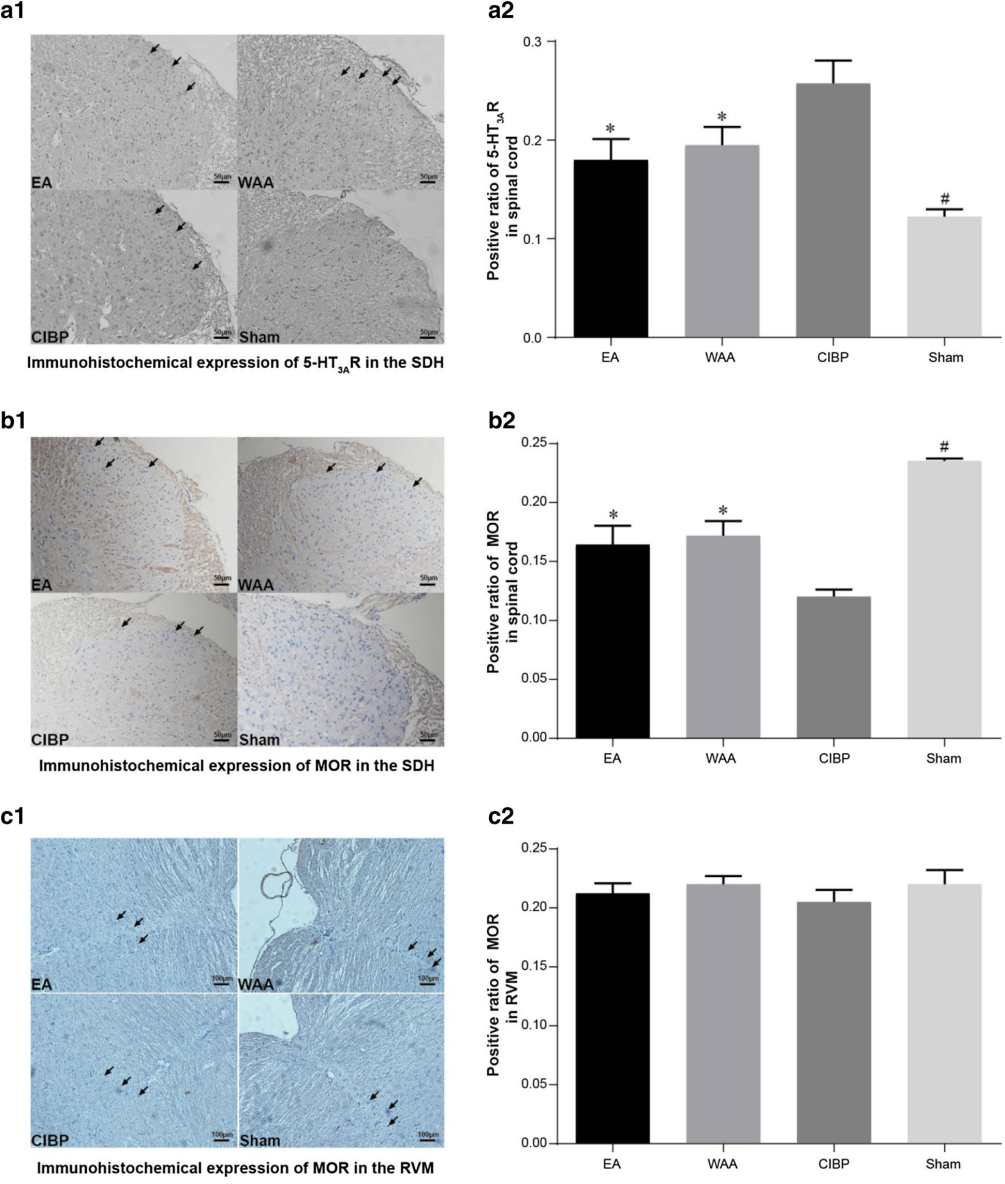
Effects of WAA on 5-HT_3A_R and MOR immunohistochemical expression. Values are mean ± standard deviations (*n* = 4) of positive proportion. *P < 0.05 vs. CIBP group; ^#^*P *< 0.05 vs. model groups (CIBP, EA, and WAA groups). *CIBP* cancer-induced bone pain, *EA* electroacupuncture, *WAA* wrist–ankle acupuncture, *5-HT*_*3A*_*R* 5-hydroxytryotamine type 3A receptor, *MOR* μ-opioid receptor, *SDH* superficial layers of the dorsal horn, *RVM* rostral ventromedial medulla

5-HT_3A_R and MOR in spinal cord are mainly located in the in the superficial layers of the dorsal horn (SDH), where nociceptive stimuli are expressed. The positive ratios of 5-HT_3A_R in the spinal cords of the three model groups (CIBP, EA, and WAA groups) were substantially higher than in the sham group (*P *< 0.05). Among the three model groups, the positive ratios of 5-HT_3A_R in the EA and WAA groups were significantly lower than that in the CIBP group (*P *< 0.05), but no significant difference was found between the EA and WAA groups (*P *> 0.05), as shown in Fig. 6a1, 2.

The positive ratios of MOR in the spinal cord of the three model groups (CIBP, EA, and WAA groups) were substantially lower than in sham group (*P *< 0.05). Among the three model groups, the positive ratios of MOR in the EA and WAA groups were significantly higher than that in the CIBP group (*P *< 0.05), but no significant difference was found between the EA and WAA groups (*P *> 0.05), as shown in Fig. 6b1, 2.

The staining of MOR in the RVM showed no difference among the four groups (*P *> 0.05; Fig. 6c1, 2).

#### ELISA results of 5-HT, β-EP, EM-1, and EM-2

**Fig. 7:**
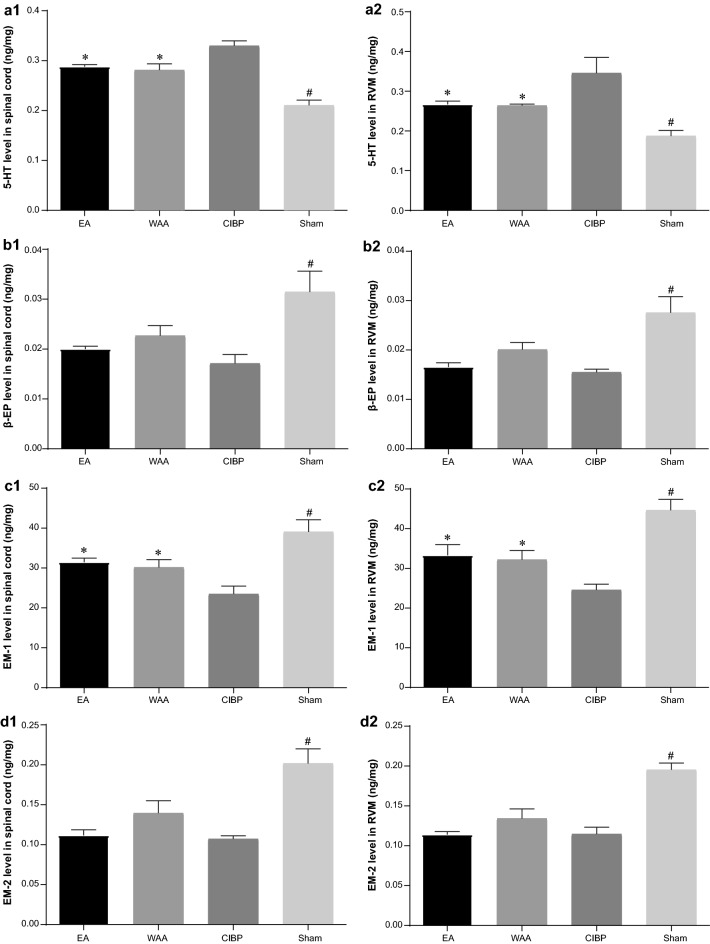
Effects of WAA on 5-HT, β-EP, EM-1, and EM-2 levels. **a1** Effects of WAA on 5-HT level in spinal cord; **a2** Effects of WAA on 5-HT level in RVM; **b1** Effects of WAA on β-EP level in spinal cord; **b2** Effects of WAA on β-EP level in RVM; **c1** Effects of WAA on EM-1 level in spinal cord; **c2** Effects of WAA on EM-1 level in RVM; **d1** Effects of WAA on EM-2 level in spinal cord; **d2** Effects of WAA on EM-2 level in RVM. Values are mean ± standard deviations (*n* = 4) of positive proportion. *P < 0.05 vs. CIBP group; ^#^*P *< 0.05 vs. model groups (CIBP, EA, and WAA groups). *CIBP* cancer-induced bone pain, *EA* electroacupuncture, *WAA* wrist–ankle acupuncture, *5-HT* 5-hydroxytryotamine, *β-EP* β-endorphin, *EM-1* endomorphin-1, *EM-2* endomorphin-2, *RVM* rostral ventromedial medulla

After the rats were killed, the levels of 5-HT, β-EP, EM-1, and EM-2 within the spinal cord and RVM were detected by ELISA. Compared with the CIBP group, the levels of 5-HT were significantly decreased by treatment with EA and WAA (*P *< 0.05, Fig. 7a1, 2). Treatment of EA and WAA also resulted in a significant increase in EM-1 levels compared with the CIBP group (*P *< 0.05, Fig. 7c1, 2). The expression levels of β-EP and EM-2 were both significantly decreased in the three model groups (CIBP, EA, and WAA groups) compared with the sham group (*P *< 0.05), but no significant difference was found among the model groups (*P *> 0.05, Fig. 7b1, 2 and d1, 2), although a trend of an increasing effect of EA and WAA on the decreased levels induced by cancer pain was observed.

## Discussion

Cancer-related pain resulting from bone metastasis has become the most common physical symptom in cancer patients that affects quality of life extremely. A rat model with site-specific metastases and similar pain symptoms to those of clinical patients, developed by Walker 256 mammary cancer cells injection into the tibial medullary cavity, is widely used for the study of cancer pain [35, 43]. The resultant tumor formation leads to bone trabecular degradation and site-specific nocifensive behavior formation. This model has closely mimicked the human pathophysiology of CIBP, involving inflammatory, neuropathic, and tumorigenic components, and revealed endogenous effectors of several targets in the periphery and at the levels of spinal cord and brain [44].

Consistent with previous reports [25, 35], it was observed in our experiment that intra-tibial injection of Walker 256 mammary cancer cells successfully led to progressive weight loss, cancer-related bone destruction, and elevated osteoclast activity. Our study also demonstrated increased pain thresholds in the CIBP rats after EA or WAA stimulation, attenuating hyperalgesia induced by bone metastasis, as shown by EA or WAA treatment that significantly increased the ipsilateral 50% PWT to the cancer cell inoculation compared with the sham control (*P *< 0.05). Analysis of the difference in analgesic effects between EA and WAA demonstrated that WAA produced a fast analgesic effect while EA (2/100 Hz) showed a comparatively slow analgesic potency (Fig. 3b). The application of WAA requires retaining needle for a long time, and the continuous stimulation may result in analgesic substances releasing earlier than EA, the mechanisms of which are still unknown and require further investigation. On day 15, ipsilateral 50% PWT increased sharply in EA group, since electroacupuncture of 2/100 Hz can inhibit hyperalgesia and hypersensitivity caused by neuropathic pain, and stimulate the cumulative effect of multiple stimuli [45].

CIBP involves both inflammatory and neuropathic mechanisms, and the descending modulation in central nervous system may take a crucial role in the perception of such a chronic pain state. The RVM is one major final output of this endogenous descending modulation and takes part in persistent nociceptive transmission between the spinal cord and brain [46]. The RVM–spinal cord is a specific centrifugal pathway that either potentiates (descending facilitation) or suppresses (descending inhibition) nociceptive transmission [46–48]. Multiple transmitters and receptors in the RVM–spinal cord pathway are involved in the descending facilitation/inhibition system [48].

It has been systemically investigated that upregulation of 5-HT levels in the spinal cord by the descending pain facilitatory system participates in the development of CIBP [20, 31]. 5-HT also exists in RVM neurons, and its increasing expression in RVM of CIBP rats has been reported in the previous study [20]. The main receptor subtype of 5-HT concentrated in SDH is 5-HT_3A_R, which is located on primary afferent C fiber terminals [49]. Activation of serotonin-positive neurons in the RVM leads to the descending of 5-HT to the spinal cord and then binding to 5-HT_3A_R.

Our ELISA results suggest that 5-HT release was increased in both spinal cord and RVM of CIBP rats, and both EA and WAA counteracted the cancer-driven upregulation of 5-HT (*P *< 0.05) in spinal cord and RVM. Western blotting and immunohistochemistry results demonstrated that 5-HT_3A_R in the spinal cord was upregulated in the CIBP group, and both EA and WAA counteracted this cancer-driven upregulation (*P *< 0.05).

RVM neurons of the descending pain inhibition system expressing MOR contribute to the maintenance and perception of neuropathic pain [50]. Selective blocking of descending facilitation-targeting MOR-positive neurons in the RVM effectively reduced the nociceptive hypersensitivity of CIBP rats [51]. Mice with *MOR* gene knockout showed no response to morphine [52]. The main endogenous ligands of MOR are EM (including EM-1 and EM-2) and β-EP. EM1 and EM2 display potent opioid activity and bind to MOR with high selectivity and affinity, and thus they are regarded as endogenous specific ligands for MOR [53–56]. The body produces β-EP, a peptide with a stronger analgesic effect than morphine, to suppress early pain, but as the pain continues the secretion of β-EP decreases gradually. Like EM, β-EP exerts its actions via MOR.

The release of MOR, as well as EM1, EM2, and β-EP, both in the spinal cord and in the RVM, was observed in our study. MOR was downregulated only in the spinal cord (SDH) in the CIBP group, and both EA and WAA counteracted this cancer-driven downregulation (*P *< 0.05), while no significant difference of MOR in the RVM was identified among the four groups. EA or WAA also counteracted the cancer-driven downregulation of EM-1 (*P *< 0.05) in the RVM and spinal cord. β-EP and EM-2 in the RVM and spinal cord were decreased remarkably in the CIBP group, but neither EA or WAA showed a significant effect on β-EP and EM-2, although a trend of increasing effect was observed (Fig. 7).

Considering these results, the anti-nociceptive effect of acupuncture is related to the facilitation/inhibition of 5-HT and MOR in the RVM–spinal cord pathway, which could be the target of analgesic treatment. Zhang et al. [29] found RVM MORs contributed to EA anti-hyperalgesia in a rat model with inflammatory pain. RVM MORs are mainly expressed in γ-aminobutyric acid (GABA) neurons, and GABA(A) receptor in the RVM is located on 5-HT neurons. The prolonged tail-flick latency induced by micro-injection of MOR agonist in the RVM was antagonized by intrathecal pretreatment with 5-HT antagonist [30]. One of the underlying mechanisms of the decrease in hyperalgesia after treatment with WAA and EA observed in our study may be that the decreased expression of 5-HT in the RVM inhibited pain facilitation, reducing 5-HT levels in the spinal cord, and downregulating 5-HT_3A_R activation.

Another mechanism may be related to the expression of MOR in the descending pain inhibition system. In our study, the decreased MOR in the spinal cord induced by CIBP was significantly increased by EA or WAA treatment. But MOR in the RVM did not decrease significantly after cancer cell inoculation, inconsistent with previous reports [57]. This may be because CIBP had not yet exerted a significant effect on the RVM due to the limited observation time. However, an increase in EM-1 in both the RVM and spinal cord was observed after WAA and EA treatment, contributing to the analgesic effects of WAA and EA. However, WAA and EA did not reverse the decrease in β-EP and EM-2 in the RVM and spinal cord in CIBP rats, inconsistent with other studies on acupuncture analgesia [14, 58–60]. Although we did not identify a statistically significant effect of EA or WAA on β-EP and EM-2, a trend of an increasing effect of EA and WAA was observed (Fig. 7). The reason for this may be connected to the small sample size that can hardly produce a statistically significant result.

In general, our findings of the present study indicated that mechanical hyperalgesia of CIBP rats could be suppressed by both EA and WAA, and WAA had a faster analgesic potency. Moreover, the analgesic mechanism of WAA in the CIBP rats may be the same as that of EA. The effect of WAA in raising the pain threshold of CIBP rats may be achieved by inhibiting the expression of 5-HT in the spinal cord and RVM, inhibiting the expression of 5-HT_3A_R in the spinal cord, increasing the expression of MOR in the spinal cord, and increasing EM-1 in the spinal cord and RVM; it is not clear whether any effects of WAA on the expression of MOR in the RVM and the expression of β-EP and EM-2 in the RVM and spinal cord exist.

Nevertheless, there are some limitations to our study. A statistically significant effect of EA or WAA on β-EP and EM-2 was not observed due to the small sample size, and the effect of WAA and EA on the expression of MOR in RVM of CIBP rats was not observed because of the limited experimental time, which will be extended in our future studies. The results of this animal experiment may not be able to be generalized to humans.

## Conclusions

WAA, a special acupuncture therapy performed only on the wrist and ankle, could significantly alleviate mechanical hyperalgesia in CIBP rats compared with EA, at least in part, by regulating of the descending pain-modulating system. This mechanism can be explained as suppressing the expression of 5-HT and 5-HT_3A_R, and increasing the expression of MOR and its ligand EM-1 in the RVM–spinal cord pathway. Furthermore, WAA produced a quicker analgesic effect than EA, the mechanisms of which may be related to retaining needle of WAA but require further investigation. WAA has been widely accepted for its ease of use and its remarkable effect in pain relief. The data from the present study provide experimental support for future research.

## Data Availability

The datasets used or analyzed during the current study are available from the corresponding author on reasonable request.

## Abbreviations

WAA: wrist–ankle acupuncture
EA: electroacupuncture
CIBP: cancer-induced bone pain
MHT: mechanical hyperalgesia threshold
PWT: paw withdrawal threshold
RVM: rostral ventromedial medullar
5-HT_3A_R: 5-hydroxytryotamine type 3A receptor
MOR: *μ*-opioid receptor
β-EP: β-endorphin
EM: endomorphin
ST36: Zusanli
BL60: Kunlun
ANOVA: one-way analysis of variance
